# The type of leg lost affects habitat use but not survival in a non‐regenerating arthropod

**DOI:** 10.1002/ece3.7879

**Published:** 2021-07-14

**Authors:** Ignacio Escalante, Damian O. Elias

**Affiliations:** ^1^ Department of Environmental Sciences, Policy, & Management University of California ‐ Berkeley CA USA; ^2^ Present address: Behavioral & Molecular Ecology Group Department of Biological Sciences University of Wisconsin – Milwaukee Milwaukee WI USA

**Keywords:** autotomy, functional morphology, harvestmen, opiliones, sensory perception

## Abstract

Finding shelter and surviving encounters with predators are pervasive challenges for animals. These challenges may be exacerbated after individuals experience bodily damage. Certain forms of damage arise voluntarily in animals; for instance, some taxa release appendages (tails, legs, or other body parts) as a defensive strategy (“autotomy”). This behavior, however, may pose long‐term negative consequences for habitat use and survival. Additionally, these putative consequences are expected to vary according to the function of the lost body part. We tested the effects of losing different functional leg types (locomotor or sensory) on future habitat use and survival in a Neotropical species of *Prionostemma* harvestmen (Arachnida: Opiliones) that undergo frequent autotomy but do not regrow limbs. Daytime surveys revealed that both eight‐legged harvestmen and harvestmen missing legs roosted in similar frequencies across habitats (tree bark, mossy tree, or fern), and perched at similar heights. Mark–recapture data showed that harvestmen that lost sensory legs roosted in tree bark less frequently, but on mossy trees more frequently. On the contrary, we did not observe changes in habitat use for eight‐legged animals or animals that lost locomotor legs. This change might be related to sensory exploration and navigation. Lastly, we found that recapture rates across substrates were not affected by the type of legs lost, suggesting that leg loss does not impact survival. This potential lack of effect might play a role in why a defensive strategy like autotomy is so prevalent in harvestmen despite the lack of regeneration.

## Graphical abstract

Some animals have the ability to release body parts to escape predators. Despite immediate benefits, this behavior conveys negative long‐term consequences. We performed a field experiment in which harvestmen lost either sensory or locomotor legs. We found that only animals that lost sensory legs changed their habitat use. However, the survival of these arachnids was not impacted by leg loss. Overall, our findings highlight how animals deal with the ecological consequences of bodily damage.

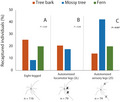

## 1 INTRODUCTION

Finding shelter and surviving encounters with predators are constant challenges for animals, and those challenges may be exacerbated when animals have a compromised body condition (Fleming et al., 2007; Stoks, 1999). Such body conditions can be physiological in nature and caused by a lack of food or energetic reserves, disease, high parasitic load, or pathogen exposure (Goodman & Johnson, 2011; Johnson et al., 2009; Vitz & Rodewald, 2011). Additionally, a compromised body condition can be morphological and/or mechanical. Bodily injury, for instance, includes the partial breakage of structures (e.g., teeth, antlers, wings, or fins), or the complete loss of body parts (e.g., tails, legs, or other body parts) (Combes et al., 2010; Harris, 1989; Maginnis, 2006). These forms of bodily damage have ecological consequences as they can impair an animal's ability to obtain food, find protected shelters, and survive (Cooper, 2003; Lin et al., 2017; Mukherjee & Heithaus, 2013). Bodily damage arises involuntarily in many cases. However, in certain taxa, damage occurs voluntarily (Emberts et al., 2017).

Many animals have the capacity to voluntarily release body parts, in the process known as “autotomy” (Emberts et al., 2019). Autotomy is frequently the cause of missing body parts in reptiles, amphibians, arthropods, mollusks, and echinoderms (Bateman & Fleming, 2009; Fleming et al., 2007; Gerald et al., 2017; Guedes et al., 2020). This extraordinary defensive behavior occurs when attempting to escape encounters with predators, after agonistic interactions with conspecifics, or in the case of arthropods, to survive a faulty molt (Maginnis, 2006). Autotomy might convey positive or negative consequences over different time scales. For example, in the short term, autotomy might allow escape (Emberts et al., 2017; Hoso & Shimatani, 2020; Naidenov & Allen, 2021). However, in the long term, autotomy can affect life‐history processes such as habitat use and future survival (Lin et al., 2017).

Autotomy has been shown to influence habitat use in various ways (Fleming et al., 2007). For example, lizards used more protected habitats such as crevices, higher branches, or tree hollows after tail loss (Cooper, 2003, 2007; Cooper & Wilson, 2008; Martin & Salvador, 1992). Similar patterns have been reported for dragonfly larvae after losing the caudal lamella (Stoks, 1999) and harvestmen after losing legs (Houghton et al., 2011). Additionally, the likelihood of autotomy varies across habitats, as found for lizards (Kuo & Irschick, 2016) and crabs (Johnston & Smith, 2018). Other studies have found no relationship between autotomy and habitat use. For instance, ground crickets with all of their legs or with legs missing hid inside hollow shelters in similar frequencies (Matsuoka et al., 2011).

Autotomy also affects future survival in various ways (reviewed in Fleming et al., 2007). For instance, some studies have found that animals missing an appendage can experience negative effects on survival and the ability to escape encounters with predators, as found for dragonfly larvae (Stoks et al., 1999), wolf spiders (Brown et al., 2018), grasshoppers (Miura & Ohsaki, 2014), crickets (Cross & Bateman, 2018), ants (Gilad et al., 2021), and lizards (Downes & Shine, 2001; Lin et al., 2017). In contrast, another subset of studies has found no effect. Appendage loss did not affect the longevity of stick insects (Carlberg, 1994), orb‐weaver spiders (Pasquet et al., 2011), or crickets (Bateman & Fleming, 2005), nor the future survival in damselfly larvae (Stoks et al., 1999) and grasshoppers (Ortego & Bowers, 1996). Interestingly, increased survival for individuals missing an appendage has also been found in leaf‐footed cactus bugs (Emberts et al., 2017).

In short, there is substantial variation in the effects of autotomy on habitat use and future survival. One plausible explanation for this pattern is that different taxa autotomize body parts with different functions, and some are more crucial to survival than others. Autotomized body parts function for locomotion, defense, sensory perception, food handling, feeding, or even reproduction (Emberts et al., 2018; Fleming et al., 2007; Maginnis, 2006). Importantly, appendages that serve different functions within taxa are usually morphologically different. For instance, locomotor legs in crabs are much longer and thinner than the feeding appendages (cheliped) (Prestholdt et al., 2018). Appendages used in spiders to transfer sperm (pedipalps) are smaller than their legs (Fromhage & Schneider, 2006). These differences in functional morphology make the loss of some body parts more critical than others (Emberts et al., 2019). However, studies exploring the consequences of losing multiple appendages that serve different functions in the same species are rare.

Understanding the ecological consequences of autotomy of different types of appendages requires experimentally studying an animal that frequently autotomizes different body parts that have different functions, but similar morphology. We explored a group of arthropods (Arachnida: Opiliones) that meet those criteria. *Prionostemma* harvestmen have two leg types: locomotor and sensory legs (Shultz et al., 2007). Legs from the first, third, and fourth pair are locomotor in function (Escalante et al., 2019; Sensenig & Shultz, 2006), whereas legs from the second pair are used as “antennae” to explore the environment (Shultz et al., 2007; Willemart et al., 2009), hence referred to as “sensory legs.” However, sensory legs are sometimes used for locomotion when harvestmen have lost locomotor legs (Escalante et al., 2020), and locomotor legs can also be used for sensory exploration (Pagoti et al., 2017). Both types of legs have the same general morphology, but the sensory legs are slightly longer and have a larger proportion of sensory organs (setae, slit sensilla, etc.) than the other legs (Wijnhoven, 2013; Willemart et al., 2009). Consequently, sensory legs could contribute more to sensory exploration by gathering information about the substrates’ properties compared with locomotor legs. Lastly, these harvestmen frequently perform leg autotomy (Domínguez et al., 2016; Escalante et al., 2013, 2020, 2021; Guffey, 1998; Powell et al., 2021), and neither juvenile nor adult individuals regenerate legs (Shultz et al., 2007).

In this study, we explored how autotomy of different functional leg types affects future habitat use and survival in one species of *Prionostemma* harvestmen. Losing legs that have different functions is known to have variable consequences for life‐history processes such as movement [see recent examples in harvestmen (Escalante et al., 2020), spiders (Wilshin et al., 2018), and crabs (Pfeiffenberger & Tsieh, 2021)]. Additionally, the habitats in which animals move affect their locomotor performance. The harvestmen we studied spend the day roosting in trees, ferns, and other plants, and disperse at night to forage and mate (Gnaspini & Willemart, 2004; Grether, Aller, et al., 2014; Grether & Donaldson, 2007; Wade et al., 2011). Harvestmen move faster on smooth bark than in mossy bark, an effect that is exacerbated if individuals are missing legs (Domínguez et al., 2016). The type of missing legs has shown variable effects in movement. Speed did not differ between individuals that lost locomotor or sensory legs in *Prionostemma* (Escalante et al., 2020), but *Holmbergiana weyenberghi* harvestmen moved slower if they were missing a sensory leg, compared with the ones missing a locomotor leg (Escalante et al., 2013). Lastly, leg loss can affect habitat use in harvestmen, as eight‐legged individuals of *Leiobunum* were found roosting higher in the same types of trees than individuals missing legs in an observational study (Houghton et al., 2011). Altogether, these studies suggest that the costs of using different habitats may differ between eight‐legged harvestmen, ones that lost locomotor, and ones that lost sensory legs. Losing sensory legs might impact how harvestmen sense and perceive the environment, potentially shaping the decisions of where to roost, and even impacting future survival. However, the effect of losing different types of legs has not been experimentally tested.

We first tested the hypothesis that the type of leg lost will differentially affect habitat use. Given the sensory legs’ role in perception and navigation, we considered that losing this type of leg—as opposed to losing locomotor legs—would impact roosting patterns. We surveyed harvestmen in the field and predicted that (1) harvestmen would be found roosting across substrates (tree bark, mossy tree, or fern) in different proportions depending on whether they had all eight legs, were missing locomotor, or were missing sensory legs. The three‐dimensional complexity and texture vary between these substrates, which affects the locomotor performance of *Prionostemma*; harvestmen missing two legs (of both types) moved slower on mossy tree (Domínguez et al., 2016). We also predicted that (2) eight‐legged harvestmen would be found roosting higher on plants than harvestmen found missing locomotor legs, which will perch higher than individuals missing sensory legs. This prediction assumes that roosting closer to the ground would result from the reduced ability to move and navigate after leg loss. Next, we experimentally induced autotomy of either locomotor or sensory legs and performed a mark–recapture study. We predicted that (3) harvestmen missing sensory legs would be more likely to be recaptured roosting across substrates in different proportions than they were marked, whereas no change will be detected in eight‐legged and individuals missing locomotor legs. Lastly, we expected (4) that perch height would differ between experimental leg loss conditions, in the same pattern as prediction 2.

The second hypothesis we tested was that the type of leg lost affects the future survival of harvestmen. For this, we compared the recapture rates between individuals that experimentally lost different types of legs. We predicted that (5) eight‐legged individuals would be recaptured more frequently than harvestmen that experimentally lost sensory legs, which will be recaptured more often than harvestmen that lost locomotor legs. Given that autotomy negatively affects the velocity, acceleration, and oxygen consumption of harvestmen while moving (Domínguez et al., 2016; Escalante et al., 2013, 2021; Guffey, 1999), the likelihood of escaping future encounters with predators might be affected.

## 2 METHODS

### 2.1 Study species and site

We surveyed an undescribed species of *Prionostemma* (*P*. sp.5, hereafter “*Prionostemma”*) (Opiliones: Sclerosomatidae) in a premontane tropical forest at Las Cruces Biological Station, San Vito de Coto Brus, Puntarenas, Costa Rica (8° 47’ N; 82° 57’ W; elevation: 1,200 m; area: 365 ha.). We explored habitat use and autotomy in adult harvestmen along the Jungle, Java, and Water trails. This omnivorous species is found in the understory of secondary and primary forest, in solitary and diurnal roosting aggregations (Guffey, 1999, Escalante et al., *in prep*.). We found that 77% of individuals in this population were roosting in aggregations of 3 to 16 individuals. Preliminary data suggest that aggregation size does not correlate with leg condition (Escalante et al., *in prep*.). Hence, we do not incorporate aggregation size in this study, as our main focus here is to test the effect of experimental leg loss on habitat use and future survival.

### 2.2 Field survey of autotomy and habitat use

To explore whether habitat use differed between leg conditions (prediction 1), we first did a field survey in which we exhaustively looked for harvestmen. Searching ranged from 0 to 3 m above ground using Pentax Papilio II 8.5 × 21 binoculars (Pentax Ltd), during the daytime (8:00 to 14:00 hr.), when these animals are typically roosting (Wade et al., 2011). For every animal found, we recorded which, if any, legs were missing as well as their roosting habitat. Hence, we are using the roost location (substrate) as a proxy for habitat use. Based on a pilot survey, we focused on the three habitats that *Prionostemma* primarily use: (1) tree bark (trunks, branches, crevices, and buttresses comprised mostly of smooth bark), (2) mossy trees (tree trunks and branches covered at least 50% by moss up to 3 cm tall), and (3) arborescent ferns of 7–10 cm diameter trunk and of up to 3 m tall. Lastly, to test for the leg condition‐specific differences in roosting height (prediction 2), we quantified each animal's perch height by measuring the distance from an individual's body to the ground with a measuring tape to the nearest 0.5 cm.

### 2.3 Autotomy experiment

#### 2.3.1 Experimental leg loss

In the second part of this project, we experimentally induced autotomy in a subset of the eight‐legged harvestmen (*n* = 269) to test whether the type of autotomy (locomotor versus sensory) affects habitat use (prediction 3). For this, we followed the same procedure as (Escalante et al., 2020, 2021). In brief, we held the animal by most of its legs and firmly held the base of the target femur with forceps. When we let go of all legs but the target leg the harvestmen immediately released the held leg.

**FIGURE 1:**
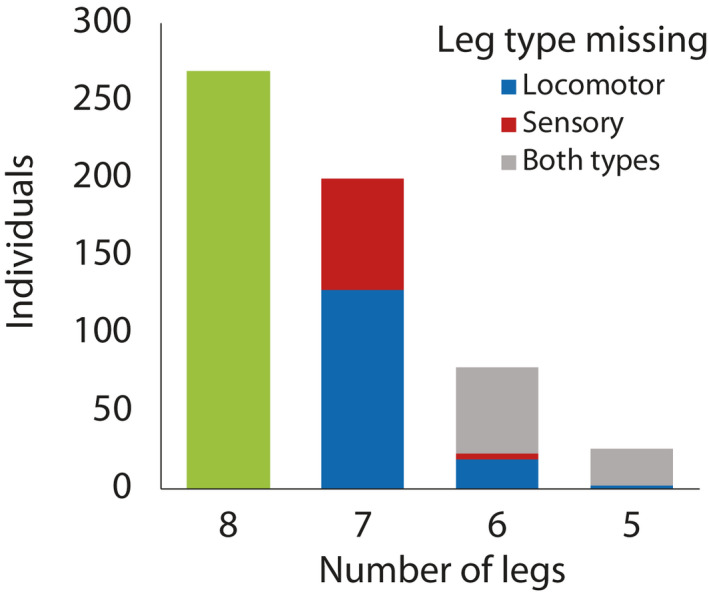
Histogram of the number of legs missing from the 573 field‐caught *Prionostemma* sp.5 harvestmen. Legend reflects the type of legs missing for individuals with <7 legs. A breakdown of each leg condition (number and types of legs missing) by substrate is shown in Table 1

We randomly assigned each individual to one of the three experimental groups: (a) individuals missing both locomotor legs of pair I (2L treatment, *n* = 79 individuals), (b) individuals missing both sensory legs (from pair II) (2S, *n* = 74), and (c) control eight‐legged individuals (C, *n* = 116), which were grabbed and held as 2L and 2S harvestmen, but without inducing autotomy. We chose these treatments to be consistent with the experimental design of previous research in which we found that losing two legs is the threshold for effects on locomotor performance (Escalante et al., 2020), and changes in oxygen consumption (Escalante et al., 2021). Additionally, missing two locomotor or sensory legs is common in our study populations (Figure 1, Table 1). Our field surveys showed that missing legs of pair IV was more common than from pair I (see Results, Appendix 1). However, as legs I are highly involved in sensory exploration, we chose to manipulate legs I to effect sensory exploration. Our field surveys also showed that missing legs from different pairs was more common than missing two legs from the same pair (see Results, Appendix 1). However, we decided to induce leg loss in the same pair to control for the potential confounding effects of losings legs of different pairs/types, which has been shown for locomotion in arthropods (including these harvestmen) (Escalante et al., 2020; Pfeiffenberger & Tsieh, 2021; Wilshin et al., 2018). We consider that our experimental treatments reflect the intensity of autotomy in the field for this species and allowed us to successfully test for the effect of different types of autotomy on habitat use and recapture rates.

**TABLE 1:** Harvestmen of *Prionostemma* sp.5 found on different substrates in field surveys according to the number and type of leg loss. Las Cruces Biological Station, Costa Rica. 2017

Leg condition		Substrate			Total
Number of legs	Type of legs missing	Tree bark	Mossy tree	Fern	
8	None	136	107	26	269
7	Locomotor	59	60	9	128
	Sensory	27	31	14	72
6	Locomotor	11	6	2	19
	Sensory	1	2	1	4
	Both	26	23	6	55
5	Locomotor (3)	0	1	1	2
	Locomotor (2) and sensory (1)	6	11	2	19
	Sensory (2) and locomotor (1)	2	2	1	5
Total		268	243	62	573

#### 2.3.2 Animals and plants marking

We marked all harvestmen over a period of five consecutive days. To do so, we used a combination of different colors of nail polish on the distal section of their opisthosoma. These marks denoted the different leg conditions (naturally occurring autotomy, experimentally induced leg loss [locomotor or sensory legs], and eight‐legged animals) and the substrate where they were found. The marks did not include the individual identity of each animal. To mark harvestmen, we held them as described above and applied a small drop of nail polish on the distal side of the opisthosoma using a small brush. Pilot observations revealed that marks persisted for at least four months (I. Escalante, *pers*. *obs*.), confirming their feasibility and suggesting they are nontoxic. Individuals were kept in a terrarium in the field for 10 min to monitor their overall condition to ensure that their behavior was unaffected. After that time, harvestmen were released at the same location in which they were collected. Lastly, we marked each tree or fern on which an animal was found by placing a small piece of flagging tape with a unique plant identification code.

#### 2.3.3 Monitoring and recapturing harvestmen

We revisited each marked plant during the daytime, every day for a period of up to 26 days. The number of resurveying days for each plant varied (24 ± 8 days, average ± standard deviation, range: 5–29 days). Because the marks did not reflect harvestmen's individual identity, we could not determine the exact number of days that each animal was searched for. We looked for harvestmen on the marked plants as well as in surrounding plants that provide the same types of substrates within a 5m radius. The active range of *Prionostemma* harvestmen is unknown. However, most of the overnight movement of *Prionostemma* is thought to be localized (Grether et al., 2014). Additionally, the recapture success found for *Prionostemma* on the same plant (15%–26%, Maginnis, 2008) allowed us to expect a reasonable sample size to test the proposed hypotheses.

When we recaptured individuals, we recorded the substrate in which it was found to test prediction 3. Also, we measured the perching height at which each individual was recaptured to test prediction 4. To avoid resampling, we took the recaptured individuals to the laboratory and kept them in a 2,000 × 50 × 50 cm terrarium with food (fruit and wet cat food) and water provided ad libitum. Hence, every harvestmen could only be recaptured once. The average period between marking and recapturing individuals was 4.6 ± 3.0 days (range: 1 – 17 days, *n* = 126 individuals). Upon completion of this project, we released harvestmen back in the forest at their approximate capture localities.

In order to confirm that *Prionostemma* harvestmen move and forage on the forest floor at night (Grether & Donaldson, 2007; Proud et al., 2012; Teng et al., 2012; Wade et al., 2011), we visited the marked roosting sites at nighttime (20:00 to 0:00 hr.) repeatedly across the study period (26 days) We confirmed that harvestmen left their roosting plant during the night. Therefore, the plant at which they were found in the following days reflected a choice of roosting sites, rather than staying on the plant that they were initially marked.

### 2.4 Experimental test of survival

To test the hypothesis that the type of autotomy affects future survival, we used recapture rates as a proxy of survival, similar to other studies [e.g., spiders (Brown et al., 2018), fishes (Runde et al., 2019), and birds (Green, 2004; Morganti et al., 2018)]. We compared recapture rates between all leg condition treatment groups (prediction 5). We calculated recapture rates as follows: (total harvestmen of a given leg condition recaptured / total harvestmen marked of that leg condition)*100. Additionally, we used the same equation to calculate the substrate‐specific recapture rate for each treatment.

Our marking method did not encode the individual identity of each harvestmen. Hence, we were unable to calculate individual recapture probability, incorporate the time since marking, or model survival (Buzatto et al., 2011; Lebreton et al., 1992; Lin et al., 2017) as a function of the type of legs lost. Despite this, comparing recapture rates between animals that lost different types of legs, as well as between substrates, allowed testing the proposed hypothesis and inferring the costs of autotomy on survival.

### 2.5 Data analysis

To determine whether the natural frequency of leg loss in the study population varied between the four leg pairs, we used a goodness‐of‐fit chi‐square test. We used another goodness‐of‐fit chi‐square to compare the frequencies of animals found across the three substrates (tree bark, mossy tree, and fern) in the field survey. We then tested if the substrates used (mossy tree, tree bark, or fern) by individuals in the field surveys differed depending on the number (8,7, or 6) and type (locomotor or sensory) of legs missing with a multinomial logistic regression. We used the substrate where harvestmen were found as the response variable, and the number of legs and the type of legs as predictor variables. We also included the interactions between the two predictor variables. For this model, we excluded the animals found with five legs, and the ones found with six legs that were missing locomotor and sensory legs, as these animals would not allow to explicitly test for the effect of leg number and type.

To examine whether perch height differed between harvestmen with different leg conditions in the field surveys (prediction 2), we ran a generalized linear model (GLM). We included perch height (in cm) as the response variable and the number of legs (8,7, or 6), leg condition (eight‐legged, missing locomotor legs, or missing sensory legs), and substrate (tree bark, mossy tree, fern) as predictor variables. We also included the interactions between the three predictor variables. For this model, we excluded animals found with 5 legs, as well as 6‐legged animals found missing locomotor and sensory legs.

With the mark–recapture data, we used a proportion chi‐square test to determine whether the number of individuals found roosting in each substrate varied within each leg condition group (missing locomotor legs [2L], missing sensory legs [2S], and eight‐legged harvestmen). This allowed us to test prediction 3. We also calculated the effect size (ES) of the odds ratio of animals of each treatment marked and recaptured on mossy tree and tree bark using the formula ES=(a*d)/(b*c), where *a* = marked on tree bark, *d* = recaptured in mossy tree, *c* = marked in mossy tree, and *b* = recaptured on tree bark. We followed (Cohen, 1988) to interpret effect sizes (0.2 as small, 0.5 as medium, and 0.8 as large). Next, we calculated the power (1 ‐ β > 0.95) of the aforementioned proportion tests using the function *pwr.2p2n.test* on the R package *pwr* (Champely et al., 2018).

To test whether perch height differed between experimental autotomy treatments (prediction 4), we ran a GLM. We used the data from recaptured individuals and included treatment (C, 2L, 2S) and substrate (tree bark and mossy tree) as predictor variables. We excluded the fern substrate from the model because we only recaptured three individuals there. We also included the treatment*substrate interaction in the model.

To infer whether experimental leg loss affected survival (prediction 5), we compared the recapture rates across the three experimental treatments with a proportion chi‐square. We used another proportion chi‐square to confirm that recapture rates were similar across substrates. The complete and raw dataset is available on Dryad. Statistical analyses were run in R (Team RC, 2019) and Microsoft Excel (version 16.43, Microsoft 2020). This research was done in compliance with institutional animal care protocols.

## 3 RESULTS

Of the 573 *Prionostemma* harvestmen surveyed, 304 (53%) were missing at least one leg (Figure 1, Table 1): 34% of individuals were missing one leg, 14% missing two legs, and 5% missing three legs (Figure 1, Table 1). As for the type of legs missing, 128 autotomized harvestmen were missing one locomotor leg, 45 were missing two or three locomotor legs, 148 were missing one sensory leg, and 9 two sensory legs (Figure 1, Table 1, Appendix 1). In the 304 autotomized harvestmen, we recorded a total of 454 legs missing (Table 1). Missing a sensory leg (from the second pair) or a locomotor leg from the fourth pair of legs occurred more often than expected by chance (37% and 30%, respectively) (*X* ^2^ = 56.3, *df* = 3, *p* < .001). In contrast, locomotor legs from the first or the third pair were missing less often than expected by chance (16% and 17%, respectively). Lastly, in the harvestmen missing two or three legs, we recorded 17 individuals that were missing both legs of the same pair (9 individuals missing both legs I, and 2 individuals were missing both legs II), and 88 individuals that were missing limbs of different pairs (Appendix 1).

### 3.1 Hypothesis 1 ‐ Habitat use and type of leg loss

#### 3.1.1 Field survey of autotomy

Harvestmen did not differ in habitat use based on their leg condition, as individuals were found across all substrates in similar proportions. First, we found that tree bark was the most frequently used substrate regardless of leg condition, with nearly half (47%) of all the 573 animals found there (*X* ^2^ = 132.5, *df* = 2, *p* < .001, Table 1). Fern was the least commonly used substrate, with only 11% of animals found there (Table 1). We did not find differences between the substrates used by eight‐legged harvestmen and harvestmen that had eight, seven, or six legs (GLM: Estimate = 0.07 ± 0.53, *p* = .90, Table 1). Additionally, habitat use did not differ between eight‐legged harvestmen and individuals missing locomotor or sensory legs (GLM: Estimate = −1.92 ± 3.89, *p* = .62, Table 1). The interaction between number and type of legs was not significant (GLM: Estimate = 0.33 ± 0.63, *p* = .59, Table 1). Therefore, we found no support for prediction 1.

**FIGURE 2:**
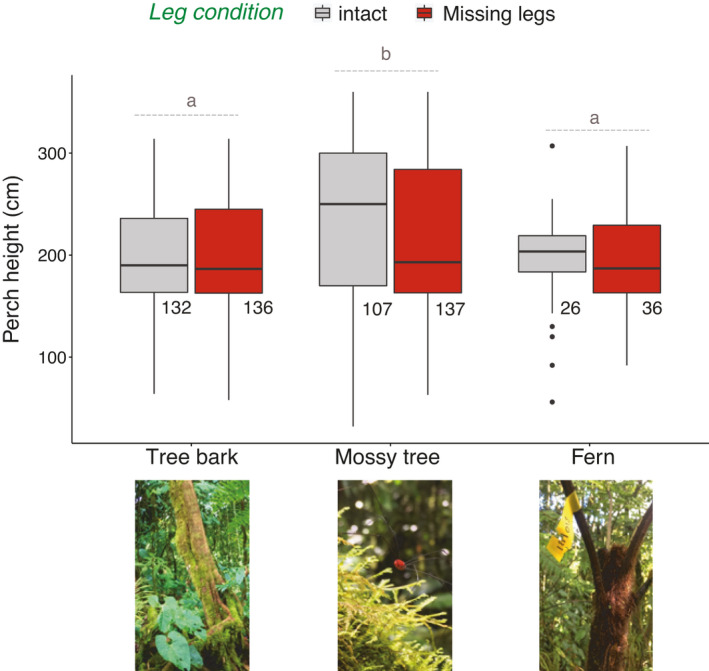
Perch heights (cm above ground) for *Prionostemma* sp.5 harvestmen in field surveys, as a function of their leg condition and the substrate where they roosted. The “missing legs” group includes all animals missing 1 or 2 legs, as well as animals missing locomotor and/or sensory legs. Those categories are pooled as they did not differ in perch height (Table 2). Boxplot center lines represent medians, with upper and lower bounds depicting ±25% quartiles. Different letters above the boxplots represent statistically significant contrasts between substrates (Table 2). Samples sizes shown below each boxplot. The picture below “mossy tree” shows *Prionostemma* sp.5, the red spherical shape is its body, from which dark gray legs extend outwards

Perch height did not differ between leg conditions. Harvestmen perched higher on mossy trees than on tree bark or ferns, but perch height did not differ between tree bark and fern (based on substrate and the post hoc comparisons; Table 2, Figure 2). This pattern did not differ based on the number or types of legs missing (Table 2, Figure 2), providing no support for prediction 2.

**TABLE 2:** Statistical results for the two models testing for differences in the perch height (in cm) in *Prionostemma* sp.5 harvestmen according to their leg condition. (I): Model results using the data from animals found in field surveys. (II): Model results using the data from animals that were recaptured after experimentally inducing them to lose legs. See Figures 2 and 4 for summary values, and Methods for further detail on the procedures. Statistical significance at the *p* < .05 level is marked with bold. Las Cruces Biological Station, Puntarenas, Costa Rica, 2017

Factor	*F*	*df*	*p*
I. Field survey			
Leg type missing (eight‐legged, missing locomotor legs, missing sensory legs)	0.26	2 – 474	.77
Number of legs (8, 7, 6)	0.01	1 – 474	.94
Substrate (tree bark ‐ mossy tree ‐ fern)	11.02	2 – 474	**<.0001**
Leg type * Substrate	0.93	4 – 474	.45
Leg number * Substrate	0.70	2 – 474	.50
Leg type * Leg number	0.01	1 – 474	.76
Leg type * Leg number * Substrate	0.13	2 – 474	.88
Post hoc comparisons			
Tree bark versus mossy tree			**<.0001**
Tree bark versus fern			.82
Mossy tree versus fern			.**003**
II. Experimental recaptures			
Treatment (Eight‐legged ‐ 2L ‐ 2S)	1.68	2 – 43	.19
Substrate (tree bark ‐ mossy tree)	14.46	1 – 43	.**0005**
Treatment * Substrate	3.76	2 – 43	.**03**
Post hoc comparisons			
Tree bark versus Mossy tree			.**001**
C ‐ tree bark versus C ‐ mossy tree			.**001**
2S ‐ tree bark versus C ‐ mossy tree			.**02**
C ‐ tree bark versus 2L ‐ mossy tree			.**03**
C ‐ mossy tree versus 2S ‐ mossy tree			.09
2L ‐ tree bark versus C ‐ mossy tree			.13
C ‐ tree bark versus 2S ‐ mossy tree			.25
2S ‐ tree bark versus 2L ‐ mossy tree			.31
C ‐ tree bark versus 2L ‐ tree bark			.44
C ‐ mossy tree versus 2L ‐ mossy tree			.70
2S ‐ mossy tree versus 2L ‐ mossy tree			.79
2L ‐ tree bark versus 2L ‐ mossy tree			.82
2S ‐ tree bark versus 2S ‐ mossy tree			.88
2S ‐ tree bark versus 2L ‐ tree bark			.93
C ‐ tree bark versus 2S ‐ tree bark			.98
2L ‐ tree bark versus 2S ‐ mossy tree			1.00

#### 3.1.2 Autotomy experiment

**FIGURE 3:**
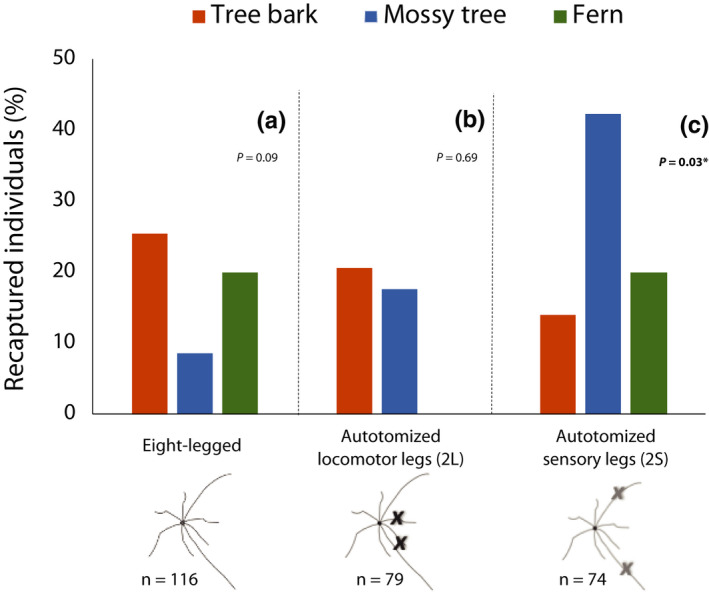
Percentage of recaptured *Prionostemma* sp.5 harvestmen across substrates. Each graph shows the percentage of individuals recaptured after experimental leg loss relative to the number of individuals marked in each treatment on that substrate. (a) Data for eight‐legged animals, (b) animals that lost two locomotor legs, and (c) individuals that lost two sensory legs. *N* = sample size of total marked individuals. Diagrams represent dorsal view of harvestmen; x indicates the experimentally autotomized legs. Leg length in the diagrams is not depicted at scale. *P* values for these analyses are shown (see Table 3 for further details on the statistical comparisons and raw values of marked and recaptured animals). Chi‐square tests of homogeneity revealed no changes in habitat use for eight‐legged animals (a) or animals that lost locomotor legs (b). Animals that lost both sensory legs (c) were recaptured significantly less frequently on tree bark—and more frequently on mossy trees—than the number in which they were initially found and marked (before experimental autotomy). Harvestmen found already missing legs were recaptured in the same proportions across substrates: tree bark (marked/recaptured): 132/40, mossy tree: 137/29, and fern: 36/5 (*X* ^2^_2_ = 5.44, *p* = .08, effect size = 0.70, Power = 0.98), overall recapture rate = 24.26% (74/305)

With the mark–recapture data, we found changes in the pattern of habitat use based on the experimentally induced leg condition. Interestingly, changes occurred in harvestmen that experimentally lost sensory legs (2S), but not in the ones that lost locomotor legs (2L) (Figure 3, Table 3). 2S harvestmen were recaptured less frequently on tree bark after leg loss and more frequently on mossy trees after leg loss (Figure 3c, Table 3). This finding supports prediction 3. On the contrary, eight‐legged harvestmen and individuals that lost 2 locomotor legs (2L) (Figure 3a and 3b, respectively, Table 3) were recaptured in similar proportions across substrates. Our estimates of effect sizes were medium–small (0.40 and 0.41) and large (0.86), based on Cohen's criteria (Cohen, 1988). Hence, we consider we had adequate power to detect differences in changes in habitat use for all treatments (Table 3).

**TABLE 3:** Left: total of *Prionostemma* sp.5 harvestmen marked and recaptured in different substrates based on their experimental leg conditions. * = The percentage of animals recaptured in each treatment reflects the relative number of recaptures by total individuals marked in each substrate (those values are plotted in Figure 3). The overall recapture rate pools substrates (tree bark, mossy tree, and fern) for each treatment and are the values used in the between‐treatment comparisons (as a proxy for survival). Recapture rates did not differ between treatments or substrates when pooling data (see Results for statistical details). Right: statistical results for the within‐treatment comparisons of marked and recaptured animals across substrates (chi‐square compared the corresponding values in bold on the left)

Event	Substrate			Total	Overall recapture rate (%)	Between substrate comparisons				
	Tree bark	Mossy tree	Fern			X2	*df*	P	Effect size	Power
Eight‐legged										
Marked	**59**	**47**	**10**	116	18.10	5.07	2	0.09	0.40	0.56
Recaptured	**15**	**4**	**2**	21						
% recaptured in that substrate*	25	9	20							
Autotomized 2 locomotor legs (2L)										
Marked	**34**	**34**	**11**	79	16.46	0.75	2	0.69	0.86	0.97
Recaptured	**7**	**6**	**0**	13						
% recaptured in that substrate*	21	18	0							
Autotomized 2 Sensory legs (2S)										
Marked	**43**	**26**	**5**	74	24.32	7.13	2	**0.03**	0.41	0.47
Recaptured	**6**	**11**	**1**	18						
% recaptured in that substrate *	14	42	20							

**FIGURE 4:**
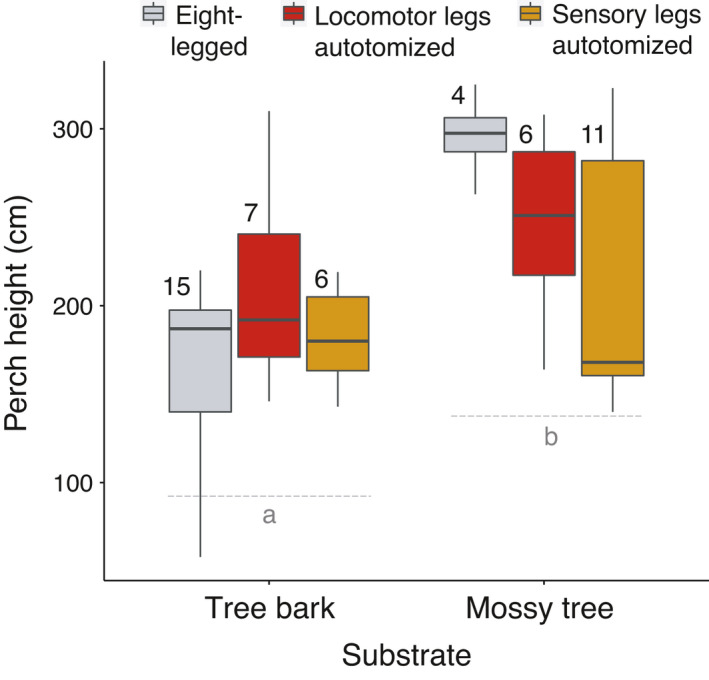
Perch heights (cm above ground) for recaptured experimentally autotomized *Prionostemma* sp.5 harvestmen as a function of their experimental leg condition and the substrate where they roosted. Boxplot center lines represent medians, with upper and lower bounds depicting ±25% quartiles. Different letters under boxplots represent statistically significant contrasts between the substrates (Table 2 for details of statistical analyses). Samples sizes shown above each boxplot

In the experiment, perch height did not differ in recaptured individuals based on the treatment groups (eight‐legged, 2L, or 2S) (based on the treatment term; Table 2). In addition, recaptured harvestmen perched higher in the mossy tree habitat than in tree bark (based on substrate term and post hoc comparisons; Table 2, Figure 4), in accordance with the data from the field survey (Table 2). Hence, prediction 4 was not met.

### 3.2 Hypothesis 2—Recapture rates and type of leg loss

Experimental leg loss did not affect recapture rates (proportion *X* ^2^ = 3.59, *df* = 3, *p* = .31, Table 3), our proxy of survival. Recapture rates did not differ between animals that experimentally lost locomotor or sensory legs, or eight‐legged individuals (Table 3). Therefore, we found no support for prediction 5. Additionally, recapture rates did not differ among substrates when data from all treatments were pooled (proportion *X* ^2^ = 5.10, *df* = 2, *p* = .08, Table 3). These two findings also support our observation that the procedure of inducing autotomy had no effect on the dispersion of harvestmen after being released.

## 4 DISCUSSION

The type of leg lost affected habitat use, but not the future survival, in *Prionostemma* harvestmen. Our field surveys showed that individuals found already missing legs (with “natural autotomy”) did not differ in roosting habitats compared with eight‐legged animals. However, for harvestmen in which we experimentally induced autotomy, losing sensory—instead of locomotor—legs resulted in a change in roosting behavior. We did not find differences, however, in perch height between eight‐legged and autotomized harvestmen, in either the field survey or the experiment. Lastly, we found that recapture rates did not differ between experimental leg conditions or habitats, which does not support the hypothesis that missing appendages affects survival.

### 4.1 Autotomy and habitat use

Our hypothesis that the type of autotomy affects habitat use was partially supported by our experimental data. Harvestmen that experimentally lost sensory legs roosted in tree bark less frequently, but on mossy trees more frequently. However, in the field surveys, habitat use did not differ between harvestmen with different types of leg loss. This suggests two alternatives. First, it may take the loss of two sensory legs to shift habitat use. The magnitude of loss has been demonstrated to impact locomotion (Escalante et al., 2020, 2021). In the field, finding individuals missing both sensory legs was rare (2%). Regardless, harvestmen missing one or two sensory legs had a similar pattern of habitat use to eight‐legged individuals suggesting this is not the case (Table 1). Consequently, the difference in habitat use between individuals with natural or experimental loss of sensory legs could be driven by both the frequency, the type, and the magnitude of leg loss. Second, harvestmen might go back to their initial habitat over a timeframe that our subsequent surveys were not able to capture. In previous experiments, it was shown that harvestmen missing several legs were, over time, able to recover most of their locomotory performance (Escalante et al., 2020). Further experimental evaluations are required to test these two alternatives.

The effects of autotomy on habitat use have been shown to be variable across taxa and across time, including in the same species of lizard (Martin & Salvador, 1992). On the one hand, several studies have shown post‐autotomy changes in habitat use (Cooper, 2003; Cooper & Wilson, 2008; Delnat et al., 2017; Houghton et al., 2011; Johnston & Smith, 2018; Stoks et al., 1999). On the other hand, no effect of autotomy on habitat use has been found in other studies (Matsuoka et al., 2011 this study). Previous studies explored the loss of only one type of appendages or body part. Thus, our results are novel in experimentally demonstrating another dimension in which autotomy can change habitat use patterns: losing appendages with different functions but similar morphology.

### 4.2 Leg type and sensory perception

Harvestmen that lost sensory legs roosted on mossy trees more often—and tree bark less often—after autotomy. This might be associated with changes in sensory perception. Sensory organs are concentrated in the second pair of legs in Sclerosomatidae harvestmen (Shultz et al., 2007; Wijnhoven, 2013; Willemart et al., 2009), and sensory legs are extensively used to tap and probe the substrates (Pagoti et al., 2017; Shultz et al., 2007; Willemart et al., 2009). Thus, sensory legs likely contribute more than locomotor legs to gathering information about the textural, mechanical, and chemical properties of the habitats. Consequently, losing sensory legs can negatively affect sensory exploration, as suggested for spiders (Miller & Mortimer, 2020). We hypothesize that harvestmen missing sensory legs may have a substantial sensory impairment that drives the changes in the selection of substrates we observed.

The sensory input animals receive from the environment drives behavior, navigation, and habitat use (Carrasco et al., 2015; Sponberg & Full, 2008; Zurek & Gilbert, 2014). We speculate that eight‐legged and harvestmen missing two locomotor legs can assess and use all habitats equally. Harvestmen missing sensory legs, on the other hand, might experience difficulty in navigating the habitats given their potentially reduced sensory ability. As a consequence, harvestmen missing sensory legs might be less able to navigate a less complex, smooth habitat such as tree bark, because this habitat potentially has fewer detectable cues available (Blaesing & Cruse, 2004; Sponberg & Full, 2008). In contrast, they might be relatively better at navigating a more complex habitat (e.g., moss) because there are more cues available there. We suggest that decreased information input after autotomy made harvestmen missing sensory legs more likely to roost on mossy trees than in tree bark. An alternative explanation could be that those harvestmen roost in mossy trees more often since its greater complexity might provide more opportunities for crypsis (see the middle picture in Figure 2)  (Gnaspini et al., 2007 and references therein). An additional alternative is that the critical component is losing legs in a specific location of the body, in this case the second pair, regardless of their function. However, all of these alternatives are speculative, and our data does not allow us to tease them apart. Future work should experimentally test the effect of autotomy on the sensory abilities and exploratory behavior of harvestmen, as well as the potential role of crypsis in roosting site selection.

### 4.3 Ecological implications of autotomy

Changes in habitat use could also be driven by predation risk perception after autotomy (Kuo & Irschick, 2016; Pears et al., 2018). For instance, if autotomy impacts locomotion, sensory abilities, and/or habitat use (Emberts et al., 2019; Escalante et al., 2020) in ways that increase exposure, animals may choose to use more protected habitats that decrease predation risk or increase crypsis (Martin & Salvador, 1992; Stoks, 1999). The changes in habitat use we observed in harvestmen that lost two sensory legs could reflect decision‐making regarding protection. However, our recapture data suggest that predation risk does not vary across habitats. Despite these observations, the possibility of autotomy affecting risk perception or predation per se across environments should be experimentally assessed, as in (Lin et al., 2017).

### 4.4 Autotomy and future survival

Harvestmen were recaptured in similar proportions regardless of the type of leg lost. Using recapture rates as a proxy for survival (as in Brown et al., 2018; Runde et al., 2019; Green, 2004; Morganti et al., 2018), we suggest that autotomy did not affect survival. Hence, we found no support for our second hypothesis. This finding highlights how these arachnids are able to withstand the changes in body condition imposed by leg loss. Missing sensory legs did not affect recapture rates, even though it changed habitat use. Together, these findings raise two possibilities: Missing legs has no impact on survival at all, or there is behavioral and/or mechanical compensation to alleviate these costs. For example, after autotomy, harvestmen could be deciding to roost on substrates such as the mossy trees that provide more crypsis from predators, as found for other animals (Cooper, 2007; Houghton et al., 2011; Johnston & Smith, 2018; Stoks, 1999). However, our data do not allow distinguish these possibilities. Future studies—for example, mesocosm experiments controlling substrate availability and predation pressure—could examine harvestmen behavior and survival after autotomy (as Dunoyer et al., 2020). Future work could also model individual‐specific survival in order to understand the long‐term implications of appendage loss on fitness and decision‐making (or lack thereof) regarding predation risk.

Across animal taxa, findings regarding survival after having lost appendages are varied. On the one hand, losing legs does not affect the longevity or future survival of different arthropod taxa (Bateman & Fleming, 2009; Fromhage & Schneider, 2006, this study). On the other hand, other studies show that appendage loss decreases future survival (Brown et al., 2018; Downes & Shine, 2001; Lin et al., 2017; Miura & Ohsaki, 2014). The lack of a common pattern for bodily damage on future survival points out that these processes depend on the particular ecological contexts in which autotomy occurs, and the functional morphology of the lost appendages.

The likelihood of recapturing *Prionostemma* harvestmen is certainly affected by many factors not explored in this project. We found an overall recapture success of 22%, which falls within the range of recapture rates for this genus (15% – 16% in, Grether & Donaldson, 2007). Factors that can affect recapture rates include animals dying (predation, parasitism, desiccation, etc.), movement to non‐surveyed areas, low site fidelity, and/or large changes in the roosting habitat (i.e., forest canopy), as suggested in previous research (Buzatto et al., 2011; Grether & Donaldson, 2007; Pagoti & Willemart, 2015). Methodological constraints could also have contributed to the recapture rate, for example, the time frame of the study (26 days), the restricted sampling area, the habitat searching strategy, and the experimental induction of leg autotomy. Regardless of the combined influence of these factors, we consider that the recapture rates we found are representative and allowed us to make robust between‐treatments and between‐substrates comparisons to test for the effect of autotomy on habitat use and future survival.

### 4.5 Potential drivers of autotomy

The location of the missing legs was not random. We found that sensory legs (pair II) and the hind locomotor legs (pair IV) were missing more frequently than legs from pairs I and III. We suggest that several factors might drive this pattern. First, legs II and IV are longer than pairs I and III in this species (Escalante and Elias, *in prep*.). Longer legs might be easier for potential predators to grab. Hence, leg length might explain autotomy patterns (Maginnis, 2006). Second, legs II are used to probe the environment, which can make them more susceptible to strikes from a potential predator, or to be seen and targeted by predators. Despite anecdotal observations of wolf and wandering spiders eating *Prionostemma* harvestmen (Escalante, *pers*. *obs*.), we do not know the specific animals that impose predatory pressures. However, this clade of arachnids is predated by many cursorial arthropods and mammals during the day and nighttime (Cokendolpher et al., 2007). Third, the high predation pressures these harvestmen experience in the premontane tropical forests might also contribute to the fact that legs IV were missing more often than expected by chance. As individuals turn away from predators, legs IV would likely be the closest part of the harvestmen to a predator. Fourth, conspecific fights could result in autotomy, as found for *Jussara* harvestmen (Pagoti et al., 2017). Fifth, faulty molts can result in autotomy (Maginnis, 2006, 2008), and some leg pairs may be more likely to be autotomized than others during molting. Although harvestmen are known to hang by all four pairs of legs while molting (Gnaspini, 2007), we are not aware of any studies examining the molting process and the likelihood to autotomize specific legs. In summary, leg length and harvestmen behavior may explain the autotomy patterns we found.

## 5 CONCLUSIONS

We experimentally demonstrated that the type of leg lost affects habitat use, but not recapture rates, in Neotropical *Prionostemma* harvestmen. Only individuals that lost sensory legs changed the habitats they used. We speculate that this change might be related to sensory exploration, navigation, and predation risk. Additionally, missing legs had no effect on recapture rates, which suggests no effect on survival. Our experiment points out how animals can be robust to the effects of autotomy and that the ecological consequences of autotomy (i.e., habitat use, foraging activity, or predation risk) are minimal in this group. The potential lack of effect on survival might play a role in explaining why a defensive strategy like voluntary appendage loss is so prevalent in harvestmen even though they do not regenerate legs.

## CONFLICT OF INTEREST

The authors declare no conflict of interest or competing interests.

## AUTHOR CONTRIBUTION

**Ignacio Escalante:** Conceptualization (equal); Data curation (equal); Formal analysis (equal); Funding acquisition (equal); Investigation (equal); Methodology (equal); Project administration (lead); Resources (equal); Visualization (equal); Writing‐original draft (equal); Writing‐review & editing (equal). **Damian O Elias:** Conceptualization (equal); Data curation (equal); Formal analysis (supporting); Funding acquisition (equal); Investigation (equal); Methodology (supporting); Visualization (supporting); Writing‐original draft (equal); Writing‐review & editing (equal).

## Data Availability

Analyses reported in this article can be reproduced using the data available on Dryad here: https://doi.org/10.5061/dryad.0p2ngf1xj

## APPENDIX 1 Description of the natural leg loss patterns in *Prionostemma* sp. 5 harvestmen. The specific leg loss refers to the legs of the four pairs and whether they were missing from the left (L) or right (R) side of the body on dorsal view. Pair number summarizes the leg pairs missing in that condition. Type of leg refers to sensory (from pair II), locomotor (pairs I, III, and IV), or a combination of both. Total number of individuals adds to 573, as shown in Table 1. Patterns of frequency for different combinations of leg loss are shown in Methods and Results.

**Table:**

Number of missing legs	Specific leg(s) lost	Pair number	Types of legs missing	Number of individuals
0	NA	NA	NA	269
1	I‐L	I	Locomotor	19
1	II‐L	II	Sensory	28
1	III‐L	III	Locomotor	17
1	IV‐L	IV	Locomotor	28
1	I‐R	I	Locomotor	13
1	II‐R	II	Sensory	43
1	III‐R	III	Locomotor	14
1	IV‐R	IV	Locomotor	37
2	I‐L,II‐L	I,II	Locomotor and sensory	6
2	I‐L,IV‐L	I,IV	Locomotor	1
2	I‐L,I‐R	I,I	locomotor	1
2	I‐L,II‐R	I,II	Locomotor and sensory	3
2	I‐L,III‐R	I,III	Locomotor	1
2	I‐L,IV‐R	I,IV	Locomotor	2
2	II‐L,III‐L	II,III	Locomotor and sensory	4
2	II‐L,IV‐L	II,IV	Locomotor and sensory	6
2	II‐L,I‐R	II,I	Locomotor and sensory	1
2	II‐L,II‐R	II,II	Locomotor and sensory	4
2	II‐L,III‐R	II,III	Locomotor and sensory	4
2	II‐L,IV‐R	I,IV	Locomotor	6
2	III‐L,IV‐L	III,IV	Locomotor	1
2	III‐L,I‐R	III,I	Locomotor	1
2	III‐L,II‐R	III,II	Locomotor and sensory	1
2	III‐L,IV‐R	III,IV	Locomotor	3
2	IV‐L,I‐R	IV,I	Locomotor	3
2	IV‐L,II‐R	IV,II	Locomotor and sensory	1
2	IV‐L,IV‐R	IV,IV	Locomotor	2
2	I‐R,II‐R	I,II	Locomotor and sensory	7
2	I‐R,IV‐R	I,IV	Locomotor	1
2	II‐R,III‐R	II,III	Locomotor and sensory	5
2	II‐R,IV‐R	II,IV	Locomotor and sensory	11
2	III‐R,IV‐R	III,IV	Locomotor	4
3	I‐L,II‐L,III‐L	I,II,III	Locomotor and sensory	2
3	I‐L,II‐L,I‐R	I,II,I	Locomotor and sensory	1
3	I‐L,II‐L,IV‐R	I,II,IV	Locomotor and sensory	3
3	I‐L,III‐L,IV‐R	I,III,IV	Locomotor	1
3	II‐L,III‐L,I‐R	II,III,I	Locomotor and sensory	1
3	II‐L,III‐L,II‐R	II,III,II	Locomotor and sensory	2
3	II‐L,III‐L,IV‐R	II,III,IV	Locomotor and sensory	1
3	II‐L,IV‐L,II‐R	II,IV,II	Locomotor and sensory	2
3	II‐L,IV‐L,III‐R	II,IV,III	Locomotor and sensory	1
3	II‐L,IV‐L,IV‐R	II,IV,IV	Locomotor and sensory	1
3	II‐L,I‐R,II‐R	II,I,II	Locomotor and sensory	1
3	II‐L,I‐R,IV‐R	II,I,IV	Locomotor and sensory	1
3	III‐L,IV‐L,I‐R	III,IV,I	Locomotor	1
3	III‐L,I‐R,II‐R	III,I,II	Locomotor and sensory	1
3	III‐L,II‐R,III‐R	III,II,III	Locomotor and sensory	1
3	III‐L,II‐R,IV‐R	III,II,IV	Locomotor and sensory	2
3	IV‐L,II‐R,IV‐R	IV,II,IV	Locomotor and sensory	2
3	I‐R,II‐R,IV‐R	I,II,IV	Locomotor and sensory	2
